# Supplementary material for: A digital twin of electrical tomography for quantitative multiphase flow imaging
Source: Commun Eng. 2022 Dec 2;1:41. doi: 10.1038/s44172-022-00042-3 (PMC10955958; doi:10.1038/s44172-022-00042-3)
Supplement: Supplementary file 2 — Supplementary information [file 44172_2022_42_MOESM2_ESM.pdf]

Supplementary Information for

A Digital Twin of Electrical Tomography for Quantitative Multiphase Flow Imaging

Authors:

Shengnan Wang[1,2], Delin Hu[1], Maomao Zhang[3], Jiawang Qiu Lin[1], Wei Chen[4], Francesco Giorgio-Serchi[5], Lihui Peng[4], Yi Li[3], Yunjie Yang[1]*

[1] The SMART Group, Institute for Digital Communications, School of Engineering, The University of Edinburgh, Edinburgh, UK.

[2] College of Electrical, Energy and Power Engineering, Yangzhou University, Yangzhou, China.

[3] Tsinghua Shenzhen International Graduate School, Shenzhen, China.

[4] Department of Automation, Tsinghua University, Beijing, China.

[5] Institute for Integrated Micro and Nano Systems, School of Engineering, The University of Edinburgh, Edinburgh, UK.

*: y.yang@ed.ac.uk

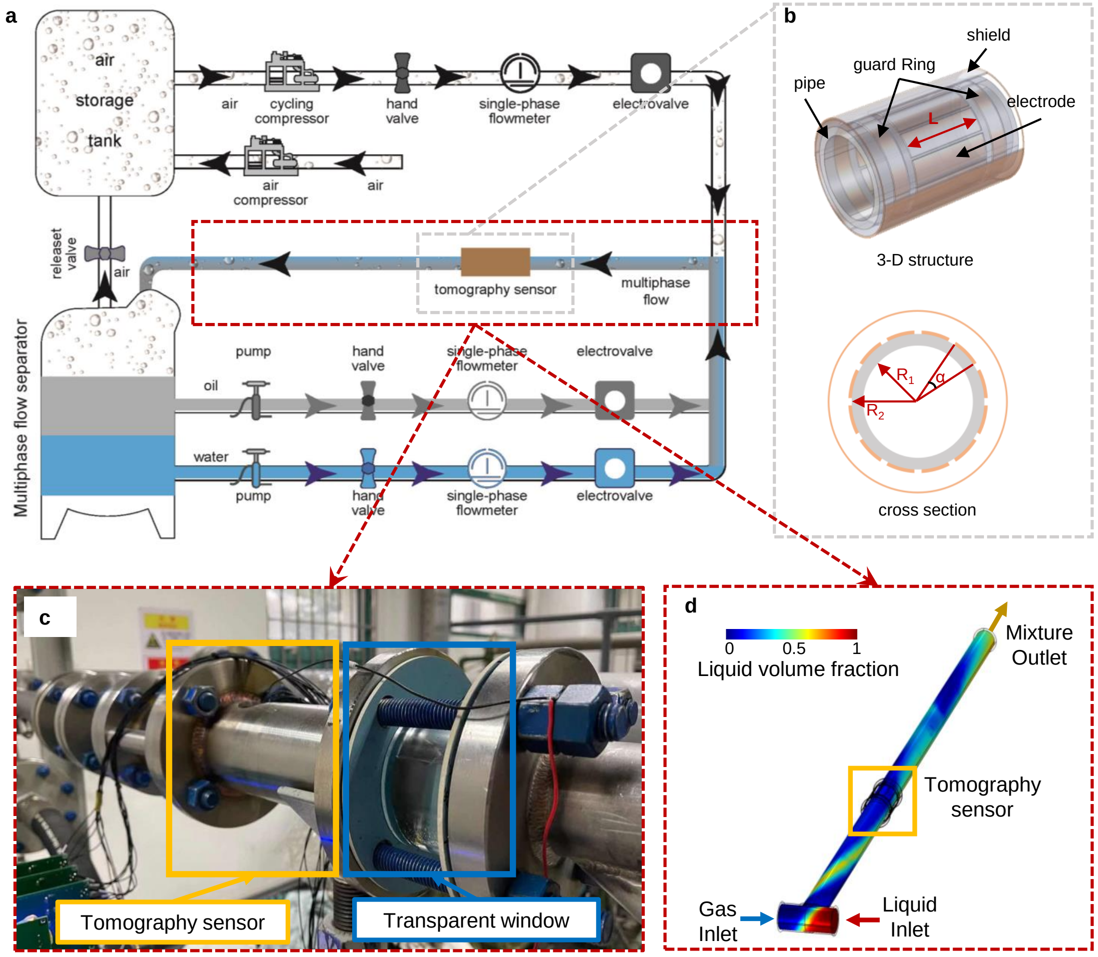

Supplementary Figure S1: The testing section of the pilot-scale multiphase flow testing facility. a Schematic illustration of the pilot-scale multiphase flow facility. **b** Schematic of the Electrical Capacitance Tomography (ECT) sensor. The ECT sensor mainly consists of twelve arc-shaped electrodes (electrode coverage angle α = 27°; electrode axial length L = 50mm), two guard rings, a grounded shield, and an insulated pipe (pipe internal diameter R_1 = 50 mm; pipe external diameter R_2 = 60 mm). **c** The physical multiphase flow imaging system. The ECT sensor with a transparent window is installed in the horizontal mixing section. The transparent window is used for visual observation during the experiment. **d** Digital representation of the physical multiphase flow imaging system. i.e., the three-dimensional fluid-electrostatic field coupling model.

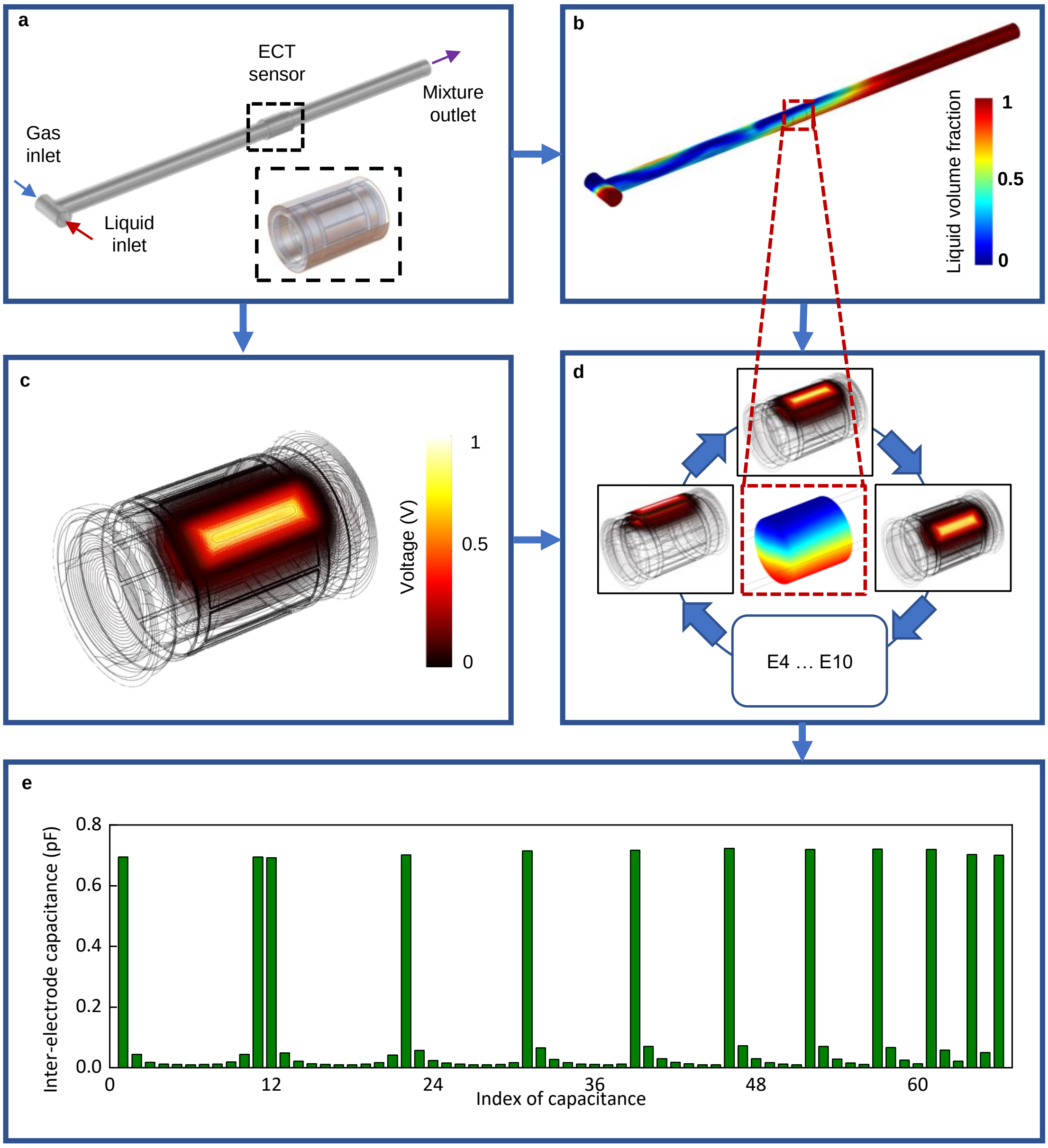

Supplementary Figure S2: Flowchart of the three-dimensional field coupling simulation. The three-dimensional Fluid-Electrostatic field Coupling Model (3D-FECM) is shown in **a**. It comprises a gas inlet, a liquid inlet, a mixture outlet, a horizontal pipe section, and a 12-electrode Electrical Capacitance Tomography (ECT) sensor. Gas and liquid are fed into the model from the two inlets, forming two-phase flows in the pipe, then flow out through the outlet. The 3D-FECM integrates a fluid field model to simulate 3D fluid field **b** and an electrostatic field model to simulate the 3D electrostatic field **c**. The two-phase flow data generated by the fluid field model can be coupled to the electrostatic field model to calculate the electric potential distribution within the ECT sensor **d**. For the 12-electrode ECT sensor, 66 independent inter-electrode capacitances **e** can be obtained in one measurement frame from the field coupling simulation.

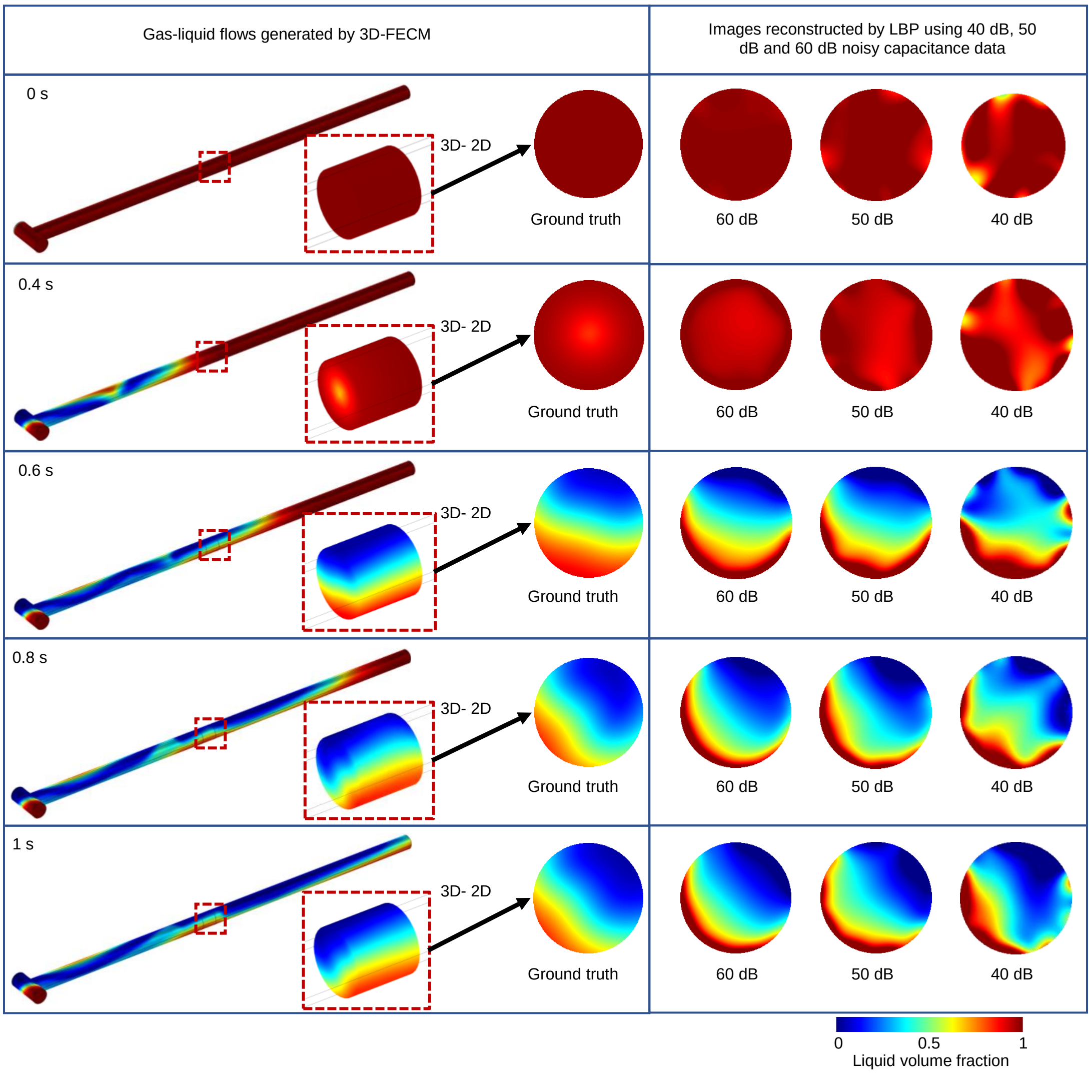

Supplementary Figure S3: A set of sequential gas-liquid flows generated by three-dimensional Fluid-Electrostatic field Coupling Model (3D-FECM) and corresponding images reconstructed by Linear Back Projection (LBP)[1] using 40 dB, 50 dB, and 60 dB noisy Electrical Capacitance Tomography (ECT) data. For the flow simulation, the inlet gas velocity is set to 1.181 m s-1; the inlet liquid velocity is set to 0.495 m s-1, and the pipe is filled with liquid in the preliminary stage. This simulation is carried out in a gravity environment. The 3D liquid phase distribution in the sensing region of ECT can be transformed into the 2D liquid phase distribution by averaging voxel-to-voxel along the axial direction of the sensor. We use the 2D liquid phase distribution as the ground truth.

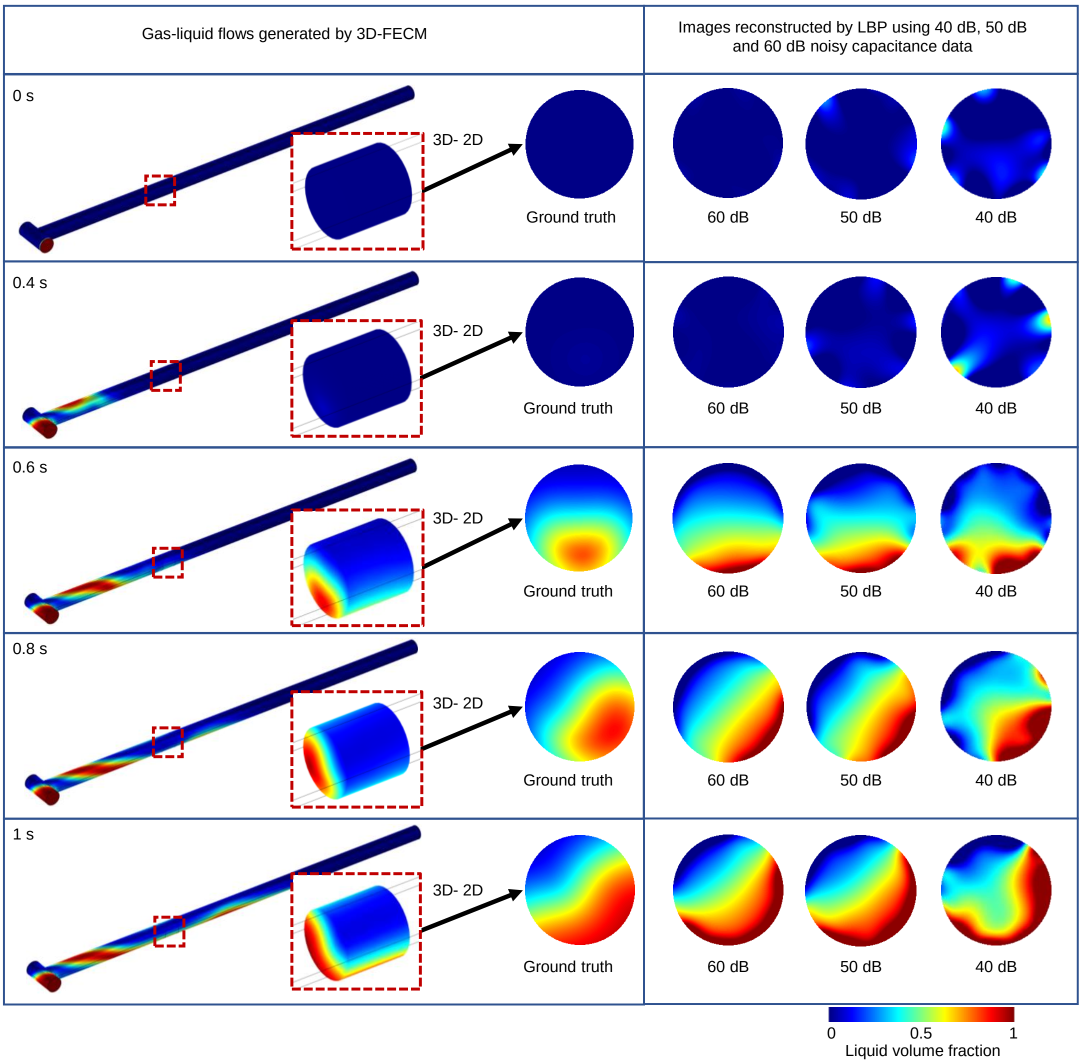

Supplementary Figure S4: A set of sequential gas-liquid flows generated by three-dimensional fluid-electrostatic field coupling model and corresponding images reconstructed by Linear Back Projection (LBP) using 40 dB, 50 dB, and 60 dB noisy electrical capacitance tomography data. This simulation is carried out under a gravity environment. At the initial stage, the pipe is filled with gas. The inlet gas and liquid velocities are set to 0.472 m s-1 and 0.708 m s-1, respectively.

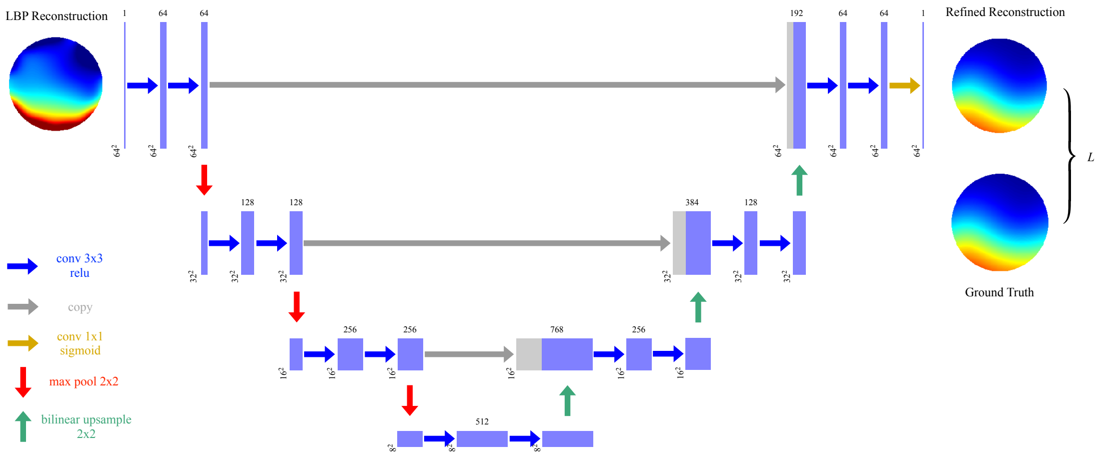

Supplementary Figure S5: The architecture of Deep Back Projection (DBP). It is a variation of the U-Net. We feed the Linear Back Projection (LBP) reconstruction that is not sufficiently accurate as the input of the network and train the network to improve the image quality, generating the final permittivity reconstruction closer to the ground truth.

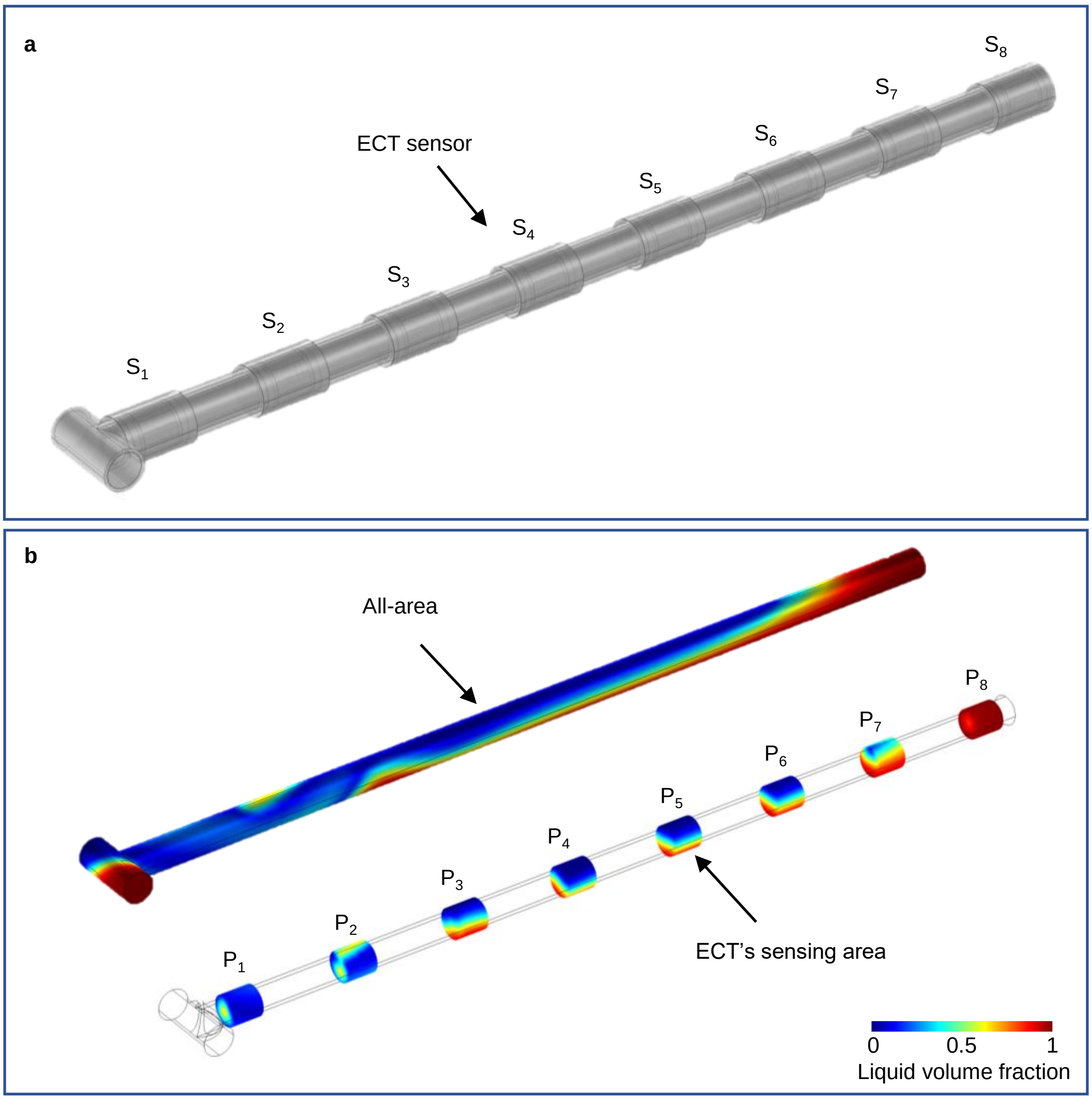

Supplementary Figure S6: Eight virtual Electrical Capacitance Tomography (ECT) sensors and their sensing regions. a Schematic diagram of the three-dimensional fluid-electrostatic field coupling model with eight identical virtual ECT sensors (named S_1, S_2, S_3, S_4, S_5, S_6, S_7, S_8, respectively) to increase sample diversity. **b** Eight dynamic liquid phase distributions in the sensing regions of the virtual ECT sensors, marked as P_1, P_2, P_3, P_4, P_5, P_6, P_7, P_8, respectively.

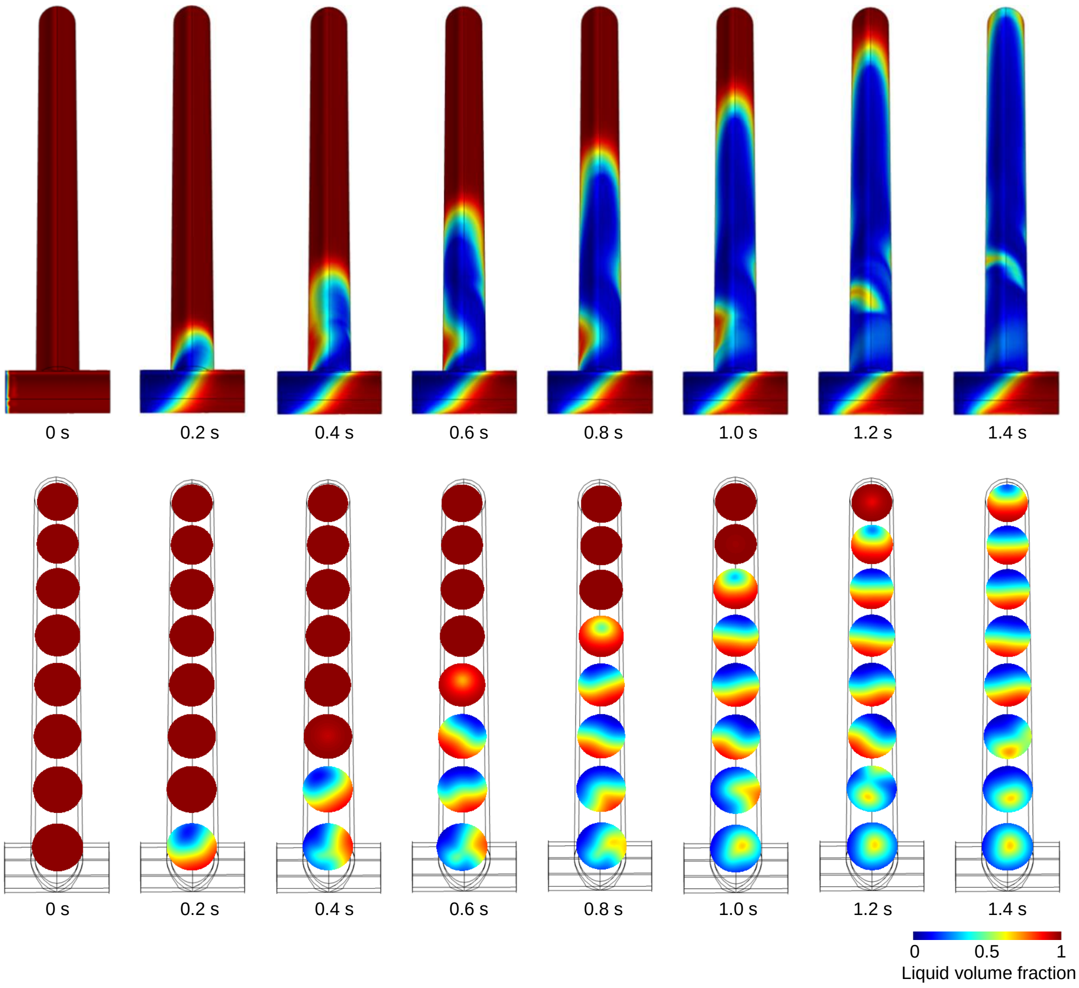

Supplementary Figure S7: A set of sequential gas-liquid flows generated by three-dimensional fluid-electrostatic field coupling model and corresponding 2D true liquid phase distributions in the sensing regions of eight virtual Electrical Capacitance Tomography (ECT) sensors. In this simulation, the pipe is initially filled with liquid. The inlet gas velocity is set to 0.709 m s-1, and the inlet liquid velocity is set to 0.425 m s-1, respectively.

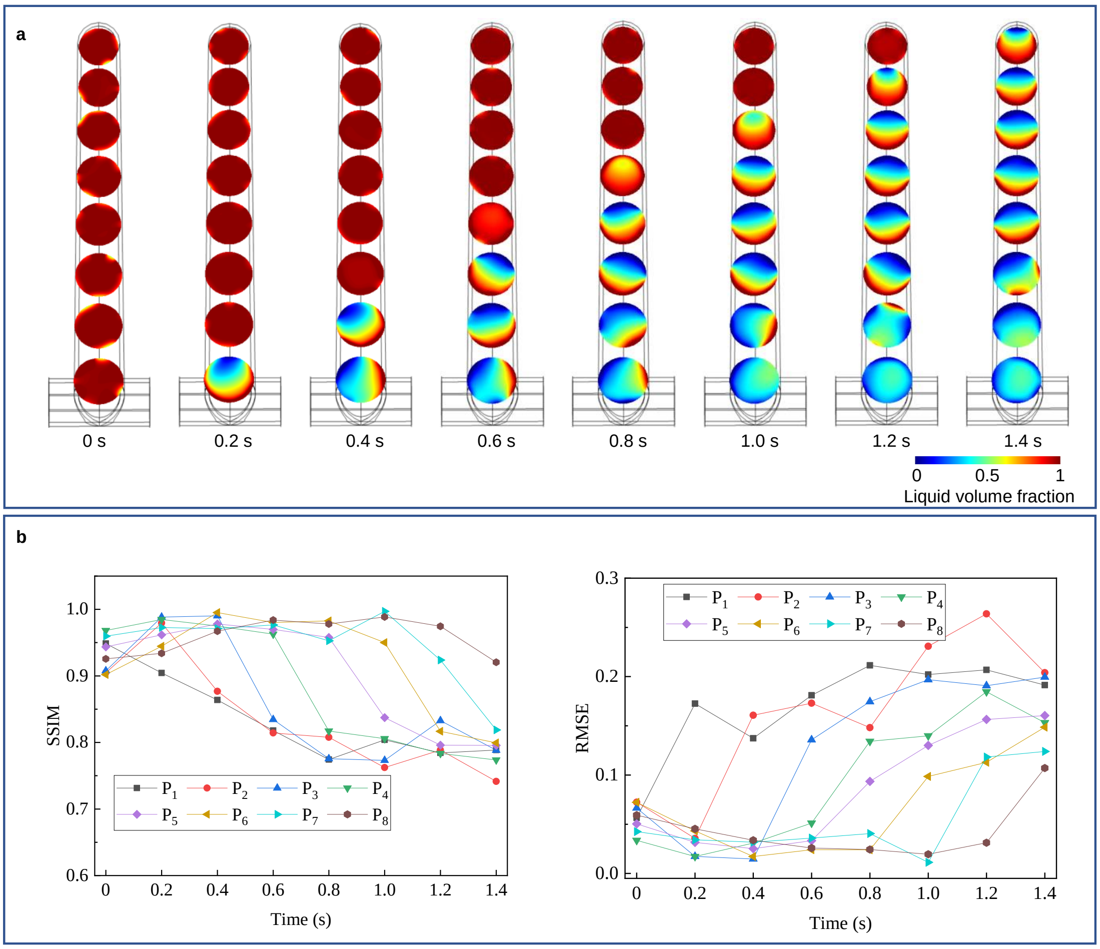

Supplementary Figure S8: Imaging of virtual gas-liquid flows across the whole horizontal section with eight virtual Electrical Capacitance Tomography (ECT) sensors by Linear Back Projection (LBP) with 50 dB Signal-Noise Ratio (SNR). a Images reconstructed by LBP for the sequential gas-liquid flows in Fig. S7. **b** The Structural Similarity Index Measure (SSIM) and Root Mean Square Error (RMSE) of the LBP results for imaging the gas-liquid flows in Fig. S7. The SSIM of each LBP result is within 0.742 to 0.997, and the RMSE ranges from 0.011 to 0.264.

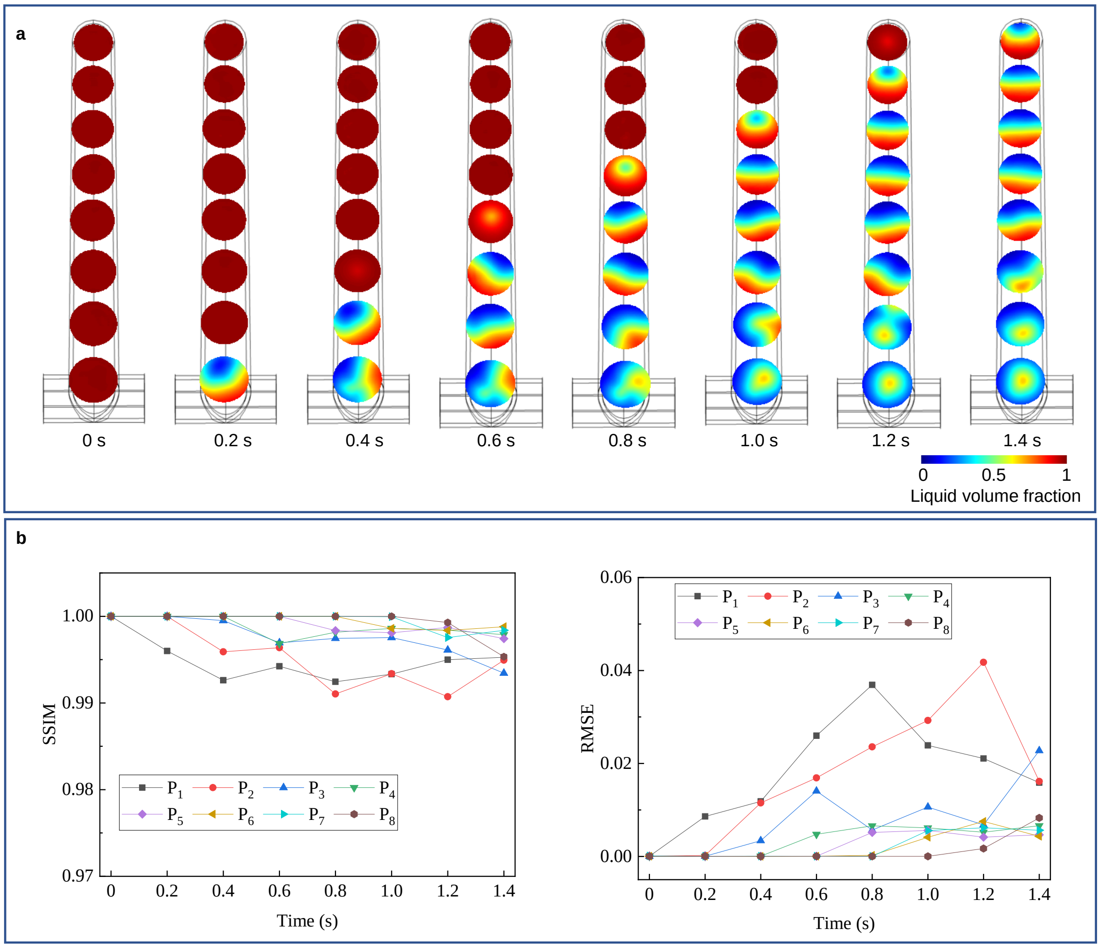

Supplementary Figure S9: Imaging of virtual gas-liquid flows across the whole horizontal section with eight virtual Electrical Capacitance Tomography (ECT) sensors by Deep Back Projection (DBP) with 50 dB Signal-Noise Ratio (SNR). a Images reconstructed by DBP for the sequential gas-liquid flows in Fig. S7. **b** The Structural Similarity Index Measure (SSIM) and Root Mean Square Error (RMSE) of the DBP results for imaging the gas-liquid flows in Fig. S7. The images reconstructed by DBP have superior quality, with the SSIMs higher than 0.991 and RMSEs lower than 0.042 for all the cases.

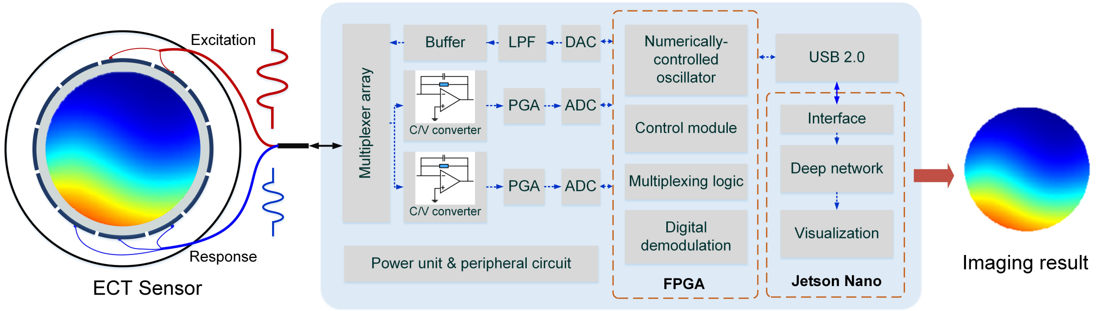

Supplementary Figure S10: The architecture of the AI-powered electrical tomography system. AC-based Electrical Capacitance Tomography (ECT) hardware structure is adopted, and the details of submodules (i.e., multiplexer array, Low Pass Filter (LPF), Programmable Gain Amplifier (PGA), and the FPGA framework) could be referred to the authors' previous work[2]. The ECT measurement module is interfaced through USB2.0 to an NVIDIA Jetson Nano, an AI computer that comprises an interface module and deep network (i.e., Deep Back Projection), and a visualization module to display the flow imaging results in real time.

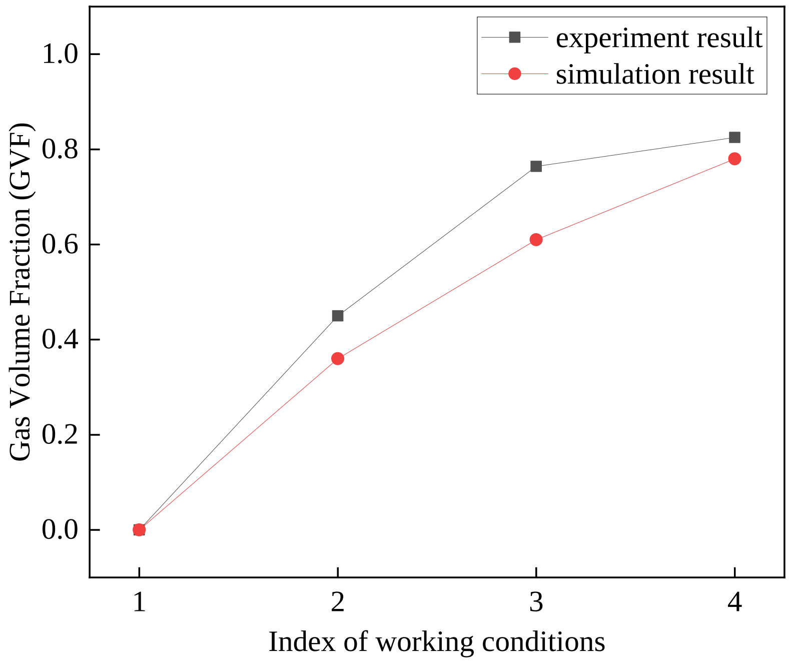

Supplementary Figure S11: Comparison of the simulation Gas Volume Fraction (GVF) (50 dB Signal-Noise Ratio) with the experiment GVF. Four working conditions are set for comparison: working condition 1 is the initial working condition where the pipe in the testing section is filled with oil; in working condition 2, the volume flow rate of air and white oil is set as 20.0 m^3 h-1 and 5.0 m^3 h-1, respectively; in working condition 3, the volume flow rate of air and white oil is set as 50.0 m^3 h-1 and 1.5 m^3 h-1, respectively; in working condition 4, the volume flow rate of air and white oil is set as 100.0 m^3 h-1 and 2.5 m^3 h-1, respectively.

Supplementary Table S1. Summary of experimental conditions. In experiments, the volume flow rate of white oil varies from 5.0 to 2.5 m^3 h-1, the air volume flow rate ranges from 20.0 to 100.0 m^3 h-1, the working pressure in the testing section is set as 0.6 MPa, and the experimental temperature is about 33 ℃.

**Index of working conditions**	**Air volume flow rate (m^3 h-1)**	**White oil volume flow rate (m^3 h-1)**	**Experimental temperature (℃)**	**Working pressure in the testing section (MPa)**
1	(Initial working condition) The pipe in the testing section is filled with white oil		33	0.6
2	20.0	5.0	33	0.6
3	100.0	2.5	33	0.6

Supplementary Table S2. Virtual dynamic experimental matrix.

(a) Summary of virtual experimental conditions

**Index**	**Initial state**	**Gravity**	**Inlet gas velocity V_{gas} (m s-1)**	**Inlet liquid velocity V_{liquid} (m s-1)**
1-60	liquid	Yes	See Table (b)	
61	gas	Yes	0.472	0.708
62	gas	Yes	1.181	0.708
63	gas	Yes	2.362	0.708
64	liquid	No	0.472	0.708
65	liquid	No	0.709	0.708
66	liquid	No	1.181	0.071
67	liquid	No	1.181	0.212
68	liquid	No	1.181	0.354
69	liquid	No	1.181	0.495
70	liquid	No	1.181	0.637
71	liquid	No	1.181	0.708
72	liquid	No	1.654	0.708
73	liquid	No	2.362	0.708

(b) Virtual experimental distribution table for the conditions of 1-60

**V_{liquid} (m s-1)**	**V_{gas} (m s-1)**					
	0.236	0.472	0.709	1.181	1.654	2.362
0.071	1	2	3	4	5	6
0.142	7	8	9	10	11	12
0.212	13	14	15	16	17	18
0.283	19	20	21	22	23	24
0.354	25	26	27	28	29	30
0.425	31	32	33	34	35	36
0.495	37	38	39	40	41	42
0.566	43	44	45	46	47	48
0.637	49	50	51	52	53	54
0.708	55	56	57	58	59	60

Supplementary References

1 Yang, W. Q. & Peng, L. H. Image reconstruction algorithms for electrical capacitance tomography. *Measurement Science and Technology* **14**, R1-R13, doi:10.1088/0957-0233/14/1/201 (2003).

2 Yang, Y. J. & Peng, L. H. A configurable electrical capacitance tomography system using a combining electrode strategy. *Measurement Science and Technology* **24**, doi:10.1088/0957-0233/24/7/074005 (2013).
