# Supplementary material for: A digital twin of electrical tomography for quantitative multiphase flow imaging
Source: Commun Eng. 2022 Dec 2;1:41. doi: 10.1038/s44172-022-00042-3 (PMC10955958; doi:10.1038/s44172-022-00042-3)
Supplement: Supplementary file 3 — Description of Additional Supplementary Files [file 44172_2022_42_MOESM3_ESM.docx]

Description of Additional Supplementary Files

**File name:** Supplementary Data 1

**Description**: Fig.2d - the inter-electrode capacitance values obtained by the coupling simulation.

**File name:** Supplementary Data 2

**Description**: Fig.3b(vii) - The SSIM and RMSE of the DBP results in (vi).

**File name:** Supplementary Data 3

**Description**: Fig.4b(iv) - Liquid volumetric concentration variations of the gas-liquid flows in a.

**File name:** Supplementary Data 4

**Description**: Fig.5a(ii) - The SSIM and RMSE of DBP results in (i). & Fig.5b(iv) - The SSIM and RMSE of DBP results in (iii).

**File name:** Supplementary Movie 1

**Description**: **Dynamic gas-liquid flows generated by 3D-FECM with different inlet liquid and gas velocities.** For Case 1, the inlet gas and liquid velocities are set to 0.472 m/s and 0.708 m/s, respectively; For Case 2, the inlet gas and liquid velocities are set to 1.181 m/s and 0.354 m/s, respectively; For Case 3, the inlet gas and liquid velocities are set to 2.362 m/s and 0.071 m/s, respectively. In these three cases, the pipe is filled with liquid in the preliminary stage, and the simulation is carried out in a gravity environment.

**File name:** Supplementary Movie 2

**Description**: **Dynamic gas-liquid flows generated by 3D-FECM with different initial conditions and different gravity environments.** The top two videos (marked Case 4 and Case 5) show the comparisons of the generated dynamic gas-liquid flows for the two cases with different initial conditions. The bottom two videos (marked Case 5 and Case 6) show the comparisons of the generated dynamic gas-liquid flows for the two cases with different gravity environments. In these three cases, the inlet gas and liquid velocities are set to 0.472 m/s and 0.708 m/s, respectively.

**File name:** Supplementary Movie 3

**Description**: **Tomographic imaging of virtual gas-liquid flow by LBP and DBP with 50 dB noisy data** (related to Fig. 3).

**File name:** Supplementary Movie 4

**Description**: **Gas-liquid flows captured by cameras under three experimental conditions.** Condition 1 is the initial working condition. In condition 1, the pipe of the testing section is filled with white oil. In condition 2, the air volume flow rate is set to 20.0 m^3^/h, and the white oil volume flow rate is set to 5.0 m^3^/h, respectively. In condition 3, the air volume flow rate is set to 100.0 m^3^/h, and the white oil volume flow rate is set to 2.5 m^3^/h, respectively (related to Fig. 4).
