# Supplementary material for: A digital twin of electrical tomography for quantitative multiphase flow imaging
Source: Commun Eng. 2022 Dec 2;1:41. doi: 10.1038/s44172-022-00042-3 (PMC10955958; doi:10.1038/s44172-022-00042-3)
Supplement: Supplementary file 1 — Yang_PR file [file 44172_2022_42_MOESM1_ESM.pdf]

nature portfolio

Peer Review File

A Digital Twin of Electrical Tomography for Quantitative Multiphase Flow Imaging

Open Access This file is licensed under a Creative Commons Attribution 4.0 International License, which permits use, sharing, adaptation, distribution and reproduction in any medium or format, as long as you give appropriate credit to the original author(s) and the source, provide a link to the Creative Commons license, and indicate if changes were made. In the cases where the authors are anonymous, such as is the case for the reports of anonymous peer reviewers, author attribution should be to 'Anonymous Referee' followed by a clear attribution to the source work. The images or other third party material in this file are included in the article's Creative Commons license, unless indicated otherwise in a credit line to the material. If material is not included in the article's Creative Commons license and your intended use is not permitted by statutory regulation or exceeds the permitted use, you will need to obtain permission directly from the copyright holder. To view a copy of this license, visit http://creativecommons.org/licenses/by/4.0/.

Reviewers' comments:

Reviewer #1 (Remarks to the Author):

The paper reports a digital twin (DT) framework of electrical tomography (ET) to address the challenge of real-time quantitative multiphase flow imaging based on non-invasive and non-radioactive technologies. The is a solid, systematic, and useful engineering work. The reviewer appreciates the effort of authors in constructing the complex system, including both hardware and software. Basically, the paper solves an inverse problem. As to novelties, it seems to the reviewer that every component of the system is existing one, but it is not trivial to put everything together. The following questions are raised for the authors to consider when revising the manuscript.
(1) The three-dimensional Fluid-Electrostatic field Coupling Model (3D-FECM) is developed as the digital representation of the physical multiphase flow imaging system. In fact, this is the forward problems solver, which will bed called by the inverse problem solver (here that is chosen as a neural network). Since the paper basically solves an inverse problem, it is strongly suggested that the term "forward problem solver" is used, instead of "digital twin (DT)".
(2) The paper solves a typical inverse problem using machine learning (ML). It seems that the paper uses ML in a brute-force (or black-box) way. In many real-world applications, data are collected by sensors automatically governed by physical laws. Some of these physical laws present well-known mathematical properties (even analytical formulas), which do not need to be learnt by training with a lot of data. It is important to answer the question of how best to combine ML with knowledge of the underlying physics as well as traditional non-ML techniques.

Reviewer #2 (Remarks to the Author):

This paper presents interesting research by proposing a digital twin of electrical tomography for quantitative multiphase flow imaging. This work is worthy of scientific attention and will arouse widespread interest in the Electrical Tomography and multiphase flow fields. Hence, I would suggest that the paper be accepted for publication. Some minor issues are provided for the authors to optimize the paper.

1. Several formulas for mixture permittivity, e.g., Maxwell Garnett, Bruggeman, et al., can be used to evaluate the equivalent permittivity of the mixture. In this paper, the authors chose the Wiener Upper Bound formula; please clarify why they chose this formula.

2. The digital twin method primarily presents 2D imaging results of the 3D flow. Why not directly output 3D results, as 3D results can provide more detailed flow information?

3. This research uses a 12-electrode ECT sensor for image reconstruction. Did the authors try ECT sensors with more electrodes, as more independent capacitance measurements can be generated for image reconstruction?

4. Some experimental validations for the simulation results can further improve the paper.

Response to Reviewers' Comments

Paper. Ref. No.: COMMSENG-22-0110-T
Title: **Digital Twin of Electrical Tomography for Quantitative Multiphase Flow Imaging**

Authors: Shengnan Wang, Delin Hu, Maomao Zhang, Jiawang Qiu Lin, Wei Chen, Francesco Giorgio-serchi, Lihui Peng, Yi Li, Yunjie Yang
The decision on the last manuscript was on **August 09, 2022**.

Referee #1's comments and our response

Comments:
The paper reports a digital twin (DT) framework of electrical tomography (ET) to address the challenge of real-time quantitative multiphase flow imaging based on non-invasive and non-radioactive technologies. The is a solid, systematic, and useful engineering work. The reviewer appreciates the effort of authors in constructing the complex system, including both hardware and software. Basically, the paper solves an inverse problem. As to novelties, it seems to the reviewer that every component of the system is existing one, but it is not trivial to put everything together. The following questions are raised for the authors to consider when revising the manuscript.

1. The three-dimensional Fluid-Electrostatic field Coupling Model (3D-FECM) is developed as the digital representation of the physical multiphase flow imaging system. In fact, this is the forward problems solver, which will better called by the inverse problem solver (here that is chosen as a neural network). Since the paper basically solves an inverse problem, it is strongly suggested that the term "forward problem solver" is used, instead of "digital twin (DT)".

Response:

We fully appreciate the reviewer's comment and suggestion.

We would like to clarify that, in this paper, the proposed Digital Twin (DT) framework of electrical tomography is not just a virtual model designed to accurately reflect a physical object (i.e., the 3D-FECM) but a system framework building upon a synergistic integration of 3D field coupling simulation, model-based deep learning, and edge computing. Here, the DT framework could be regarded as a platform allowing ET to learn the dynamic flow features in the virtual space and enable quantitative multiphase flow imaging in the physical space. 3D-FECM is only one of the critical components of the overall DT framework (see Fig. 1). Therefore, while we understand the reviewer's concern with adequate naming the proposed framework, we feel that the "forward problem solver" overlooks the role of the neural network within the learning component of the imaging process and the other components that connecting the physical system and the virtual space as a closed-loop platform. Our original choice for the usage of DT as the key terminology for our imaging technique lies precisely in the combination of training, prediction, and measurements.

The core concept of the digital twin envisages a system that couples physical entities with virtual counterparts, leveraging the benefits of both the virtual and physical environments to benefit the entire system **[Ref1]**. Current ET technology suffers from low spatial resolution

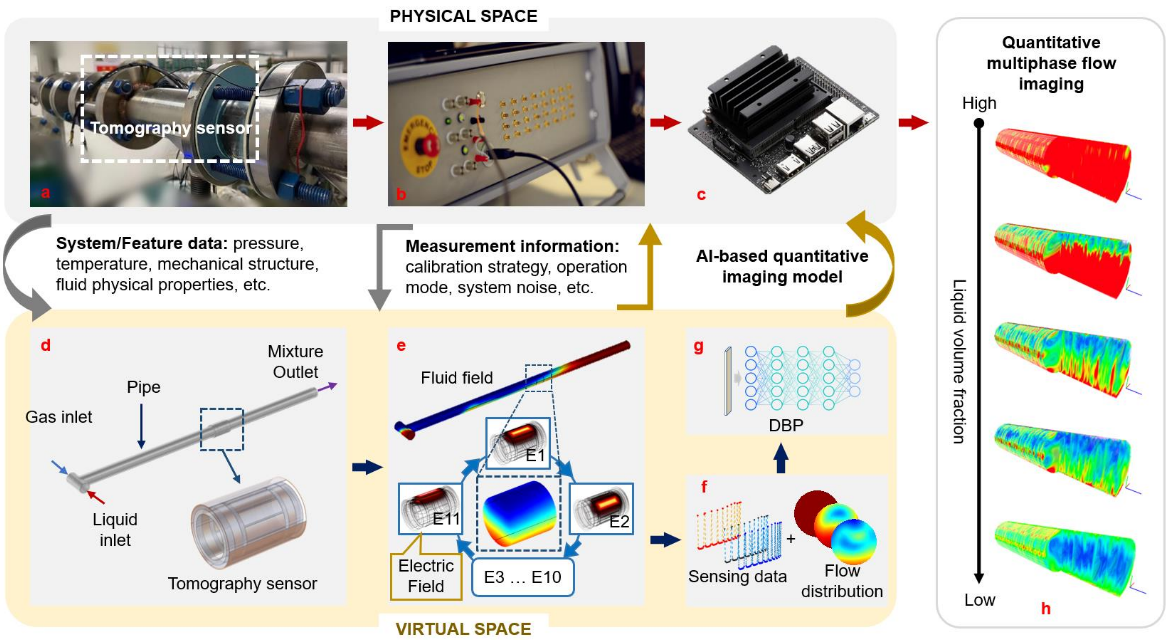

***Fig. 1. The digital twin framework of electrical tomography for quantitative multiphase flow imaging. a** The testing section of the multiphase flow facility. The system/feature data are captured via the sensors installed in the testing section. **b** The electrical tomography system. **c** The edge computer (i.e. NVIDIA Jetson Nano) implements the AI-based quantitative imaging model. **d** The 3D geometrical model of the testing section and the 12-electrode tomography sensor. **e** Schematic of the 3D fluid-electric field coupling simulation based on the model in **d**. **f** Illustration of the generated virtual tomographic sensing data and corresponding dynamic flow distributions within the sensing region. **g** The model-based deep neural network (DBP) for quantitative flow imaging. DBP is trained based on the data set in **f** and implemented in **c**. **h** The edge computer's output generates quantitative flow images and key parameters estimation based on the output of **b**.*

(around 10% of the sensor diameter) due to the nonlinear ill-posed inverse problems. Another limiting factor in dealing with multiphase flow ET imaging is the lack of access to the ground truth of the real multiphase flow for the mapping between the electrical signal and flow distribution, as well as for quantitative image quality evaluation, which is also essential in optimizing the image reconstruction algorithms of ET. The emerging DT technology has the potential to resolve these issues. Therefore, we first introduce the DT concept into the ET-based multiphase flow imaging field, aim to provide a new paradigm for quantitative multiphase flow imaging, and extend the limit of ET.

We have also modified the revised manuscript (***Supplementary Information-Figure S1***) to make our claims more straightforward.

[Ref1] Jones, D., Snider, C., Nassehi, A., Yon, J. & Hicks, B. Characterising the Digital Twin: A systematic literature review. *Cirp Journal of Manufacturing Science and Technology* **29**, 36-52, doi:10.1016/j.cirpj.2020.02.002 (2020).

Supplementary Information-Figure S1:

Change

"***Supplementary Figure S1: Digital twin of the test section of the pilot-scale multiphase flow testing facility. a** Schematic illustration of the pilot-scale multiphase*

flow facility. ***b*** *Schematic of the ECT sensor. The ECT sensor mainly consists of twelve arc-shaped electrodes (electrode coverage angle α = 27°; electrode axial length L = 50mm), two guard rings, a grounded shield, and an insulated pipe (pipe internal diameter R_1 = 50 mm; pipe external diameter R_2 = 60 mm).* ***c*** *Physical twin of the test section of the multiphase flow facility. The ECT sensor with a transparent window is installed in the horizontal mixing section. The transparent window is used for visual observation during the experiment.* ***d*** *Digital twin of the test section of the multiphase flow facility."*

To

*"**Supplementary Figure S1: The testing section of the pilot-scale multiphase flow testing facility. a** Schematic illustration of the pilot-scale multiphase flow facility.* ***b*** *Schematic of the ECT sensor. The ECT sensor mainly consists of twelve arc-shaped electrodes (electrode coverage angle α = 27°; electrode axial length L = 50mm), two guard rings, a grounded shield, and an insulated pipe (pipe internal diameter R_1 = 50 mm; pipe external diameter R_2 = 60 mm).* ***c*** *The physical multiphase flow imaging system. The ECT sensor with a transparent window is installed in the horizontal mixing section. The transparent window is used for visual observation during the experiment.* ***d*** *Digital representation of the physical multiphase flow imaging system. i.e., the three-dimensional Fluid-Electrostatic field Coupling Model (3D-FECM)."*

2. The paper solves a typical inverse problem using machine learning (ML). It seems that the paper uses ML in a brute-force (or black-box) way. In many real-world applications, data are collected by sensors automatically governed by physical laws. Some of these physical laws present well-known mathematical properties (even analytical formulas), which do not need to be learnt by training with a lot of data. It is important to answer the question of how best to combine ML with knowledge of the underlying physics as well as traditional non-ML techniques.

***Response**:*

We fully appreciate the reviewer's comment.

Two commonly recognized issues of the ECT inverse problem make it a long-standing challenge in the field. First, it is a severely ill-posed and underdetermined problem. Second, ECT has the "soft-field" effect, i.e., the sensitivity of the ECT sensor varies with the actual permittivity distribution, leading to the nonlinear mapping from the measurement to the electrical property distribution space.

In the past, many conventional model-based image reconstruction algorithms have been developed to address the ECT inverse problem. These algorithms taking advantage of the underlying physics of ECT can be generally categorized into two groups, i.e., the non-iterative algorithms, e.g., Linear Back Projection (LBP) **[Ref1]**, truncated singular value decomposition **[Ref2]** and D-bar method **[Ref3]**, and iterative algorithms, e.g., Total Variation (TV) regularization **[Ref4]**, sparse regularization **[Ref5]** and sparse representation **[Ref6]**. However, it remains an open challenge for these model-based algorithms to achieve quantitative imaging of complex multiphase flows.

In recent years, the evolution of machine learning and data-driven methods has brought opportunities to resolve such nonlinear and ill-posed inverse problems from a different perspective due to their capability to handle highly complex, dynamic, and nonlinear problems. However, it is also well-recognized that pure end-to-end learning approaches

(e.g., the 'black box' way) have led to the concerns of abandoning the underlying physical laws and over-dependence on the datasets. Model-based learning approaches or post-processing methods **[Ref7]** that try incorporating physical information into machine learning have become a prevailing branch.

In this work, we adopted the post-processing approach and designed a light-weighted network named Deep Back Projection (DBP) to demonstrate the feasibility of the whole DT framework. DBP first uses the conventional LBP algorithm to reconstruct the initial permittivity distribution and refines the LBP results with a customized U-Net. This approach takes advantage of the physical model and machine learning. In the discussion section of the paper, we also explicitly mentioned:

Main text-Discussion (Pg. 13, line 241):
It is also noteworthy that the focus of this work is the overall DT framework rather than the learning-based algorithm for ECT image reconstruction. We demonstrate that our DT framework can achieve superior performance even using simple neural architectures like DBP. We also point out that employing dedicated and more specialized neural architectures can potentially lead to a better performance at the cost of a larger training dataset and a more complex training strategy.

The dataset plays a central role in the machine learning approach and determines the network's accuracy and generalization ability. We make the first attempt to introduce the DT concept into the ET field to effectively conduct large-scale virtual experiments according to the working conditions of the multiphase flow testing facility and synthesize a virtual dataset consisting of abundant multiphase flows and corresponding ECT measurements (the virtual multiphase flow dataset is available at https://datashare.ed.ac.uk/handle/10283/4483). In the future, many other types of approaches, e.g., model-based or physics-informed deep neural networks, can be designed, trained, and evaluated using the virtual dataset.

[Ref1] Sun, B. Y., Yue, S. H., Cui, Z. Q. & Wang, H. X. A new linear back projection algorithm to electrical tomography based on measuring data decomposition. *Measurement Science and Technology* **26**, doi:10.1088/0957-0233/26/12/125402 (2015).

[Ref2] Xu, Z. *et al.* An Image Reconstruction Algorithm for a 12-Electrode Capacitively Coupled Electrical Resistance Tomography System Under 2-Electrode Excitation Strategy. *Ieee Transactions on Instrumentation and Measurement* **70**, doi:10.1109/tim.2021.3098388 (2021).

[Ref3] Hamilton, S. J., Lionheart, R. B. & Adler, A. Comparing D-bar and common regularization-based methods for electrical impedance tomography. *Physiological Measurement* **40**, doi:10.1088/1361-6579/ab14aa (2019).

[Ref4] Hinze, M., Kaltenbacher, B. & Quyen, T. N. T. Identifying conductivity in electrical impedance tomography with total variation regularization. *Numerische Mathematik* **138**, 723-765, doi:10.1007/s00211-017-0920-8 (2018).

[Ref5] Zhong, S. C. *et al.* Electrical resistivity tomography with smooth sparse regularization. *Geophysical Prospecting* **69**, 1773-1789, doi:10.1111/1365-2478.13138 (2021).

[Ref6] Ye, J. M., Wang, H. G. & Yang, W. Q. Image Reconstruction for Electrical Capacitance Tomography Based on Sparse Representation. *Ieee Transactions on Instrumentation and Measurement* **64**, 89-102, doi:10.1109/tim.2014.2329738 (2015).

[Ref7] Xiang, J. X., Dong, Y. G. & Yang, Y. J. FISTA-Net: Learning a Fast Iterative Shrinkage Thresholding Network for Inverse Problems in Imaging. *Ieee Transactions on Medical Imaging* **40**, 1329-1339, doi:10.1109/tmi.2021.3054167 (2021).

Reviewer #2's comments and our response

Comments:

This paper presents interesting research by proposing a digital twin of electrical tomography for quantitative multiphase flow imaging. This work is worthy of scientific attention and will arouse widespread interest in the Electrical Tomography and multiphase flow fields. Hence, I would suggest that the paper be accepted for publication. Some minor issues are provided for the authors to optimize the paper.

1. Several formulas for mixture permittivity, e.g., Maxwell Garnett, Bruggeman, et al., can be used to evaluate the equivalent permittivity of the mixture. In this paper, the authors chose the Wiener Upper Bound formula; please clarify why they chose this formula.

Response:

Several mixture formulas, e.g., Maxwell-Garnett, Bruggeman, and Wagner, have been studied to evaluate the equivalent permittivity of the two-component mixture **[Ref1, Ref2]**. Among them, the simplest one is the arithmetic averaging without account of mutual interaction of the components, known as the Wiener Upper Bound formula, which is usually used for dissolved inclusion; the more classical and famous ones are the Maxwell Garnett formula and the Bruggeman formula. It is also found that when the dielectric perturbation and the volume fraction of inclusion are insignificant, most formulas are good enough to evaluate the equivalent permittivity. However, no formula seems to be able to predict the effective permittivity of mixtures over the whole range of volume fractions. Comprehensively considering the fluid condition and the computing efficiency, the Wiener Upper Bound formula is selected in this paper. The ECT reconstruction results also indicated that the Wiener Upper Bound formula is suitable for evaluating the oil-gas mixture's equivalent permittivity.

[Ref1] N. T. Liu, Y. Q. Jin, "A discussion on the effective permittivity of multi-component medium derived by Maxwell-Garnett, strong fluctuation and quasicrystalline-CP modeling," *Waves in Random and Complex Media*, DOI: 10.1080/17455030.2020.1711991.

[Ref2] K. Karkkainen, A. Sihvola, and K. Nikoskonen, "Effective permittivity of mixtures: numerical validation by the FDTD method," IEEE Trans. Geosci. Remote Sens., vol. 38, no. 3, pp. 1303-1308, May 2000.

2. The digital twin method primarily presents 2D imaging results of the 3D flow. Why not directly output 3D results, as 3D results can provide more detailed flow information.

Response:

We fully agree that 3D analysis will be beneficial for multiphase flow imaging. However, this study mainly focuses on the single-layer ECT sensor with 12 electrodes (which outputs a quasi-2D image of the flow) rather than multi-layer ECT sensors that can provide 3D imaging. It is our most immediate priority in the near future to investigate the 3D effect of

different 3D ECT sensors to provide accurate 3D imaging of two-phase flows based on 3D coupling field simulation.

3. This research uses a 12-electrode ECT sensor for image reconstruction. Did the authors try ECT sensors with more electrodes, as more independent capacitance measurements can be generated for image reconstruction?

Response:

With the increase in the number of electrodes, the number of independent measurements increases, and an improved image resolution might be obtained. However, the increase in the number of electrodes might result in the following issues: (1) smaller capacitance (and larger dynamic range of measured capacitance) to be measured; (2) complicated and expensive hardware; (3) larger eigenvalues of the sensitivity matrix; and (4) a slower data acquisition rate due to more measurements to be taken.

Peng et al. **[REF1]** systematically studied the effect of the number of electrodes on the performance of ECT sensors and found that, in most cases, the ECT sensor with 12 electrodes can provide the best 2D images. Therefore, a 12-electrode ECT sensor is adopted in this research.

[Ref1] Peng, L. H., Ye, J. M., Lu, G. & Yang, W. Q. Evaluation of Effect of Number of Electrodes in ECT Sensors on Image Quality. *IEEE Sensors Journal* **12**, 1554-1565, doi:10.1109/jsen.2011.2174438 (2012).

4. Some experimental validations for the simulation results can further improve the paper.

Response:

We fully appreciate the reviewer's comment. The revised manuscript has added some details regarding validating the simulation results. (***Main text-Results; Supplementary Information-Figure S11***). As Gas Volume Fraction (GVF) is one of the key parameters describing a gas-liquid flow system, we compared the experiment GVF with the simulation GVF.

Main text-Results (Pg. 10, line 172):

We conduct gas-liquid dynamic flow imaging experiments in virtual space, in line with the physical experimental setup, as validation test case to further verify the reliability of 3D-FECM. Gas Volume Fraction (GVF) is one of the critical parameters describing a gas-liquid flow system, and we calculate the mean value of GVF for each working condition by averaging the continuous measurements in the quasi-static stage. Supplementary Figure S11 compares the GVF results between the simulation and experiment. We can see that the simulation results agree with the experiment across each working condition, and capture the temporal evolution of the mixture throughout its transient response to varying working conditions.

Supplementary Information-Figure S1:

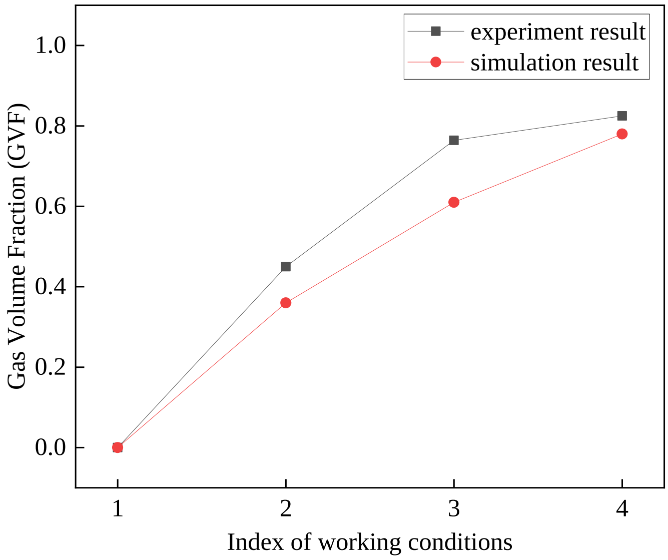

Supplementary Figure S11: Comparison of the simulation GVF (50 dB SNR) with the experiment GVF. *Four working conditions are set for comparison: working condition 1 is the initial working condition where the pipe in the testing section is filled with oil; in working condition 2, the volume flow rate of air and white oil is set as 20.0 m^3/h and 5.0 m^3/h, respectively; in working condition 3, the volume flow rate of air and white oil is set as 50.0 m^3/h and 1.5 m^3/h, respectively; in working condition 4, the volume flow rate of air and white oil is set as 100.0 m^3/h and 2.5 m^3/h, respectively.*

We would like to thank you again for the great effort of the editor and both reviewers, and we hope the revised manuscript has addressed all the concerns.

Best wishes,
Shengnan Wang, Delin Hu, Maomao Zhang, Jiawang Qiu Lin, Wei Chen, Francesco Giorgio-serchi, Lihui Peng, Yi Li, Yunjie Yang

REVIEWERS' COMMENTS:

Reviewer #1 (Remarks to the Author):

The quality of the revised manuscript is significantly improved and has adequately addressed my questions.

Reviewer #2 (Remarks to the Author):

The authors have made substantial revisions to this paper. I have no further comments.

Response to Reviewers' Comments

Paper. Ref. No.: COMMSENG-22-0110A
Title: **A Digital Twin of Electrical Tomography for Quantitative Multiphase Flow Imaging**

Authors: Shengnan Wang, Delin Hu, Maomao Zhang, Jiawang Qiu Lin, Wei Chen, Francesco Giorgio-serchi, Lihui Peng, Yi Li, Yunjie Yang
The decision on last manuscript was on **November 01, 2022**.

Reviewer # 1 (Remarks to the Author)
Comments:
The quality of the revised manuscript is significantly improved and has adequately addressed my questions.

Reviewer # 2 (Remarks to the Author)
Comments:
The author have made substantial revisions to this paper. I have no further comments.

Response to Reviewers:

We'd like to thank again the great effort of all reviewers.

Best wishes,
Shengnan Wang, Delin Hu, Maomao Zhang, Jiawang Qiu Lin, Wei Chen, Francesco Giorgio-serchi, Lihui Peng, Yi Li, Yunjie Yang
